# Effect of a 3-Month Modified, High-Quality Take-Home Ration on Linear Growth and Body Composition in Rural South Indian Infants Aged 6–9 Months: A Cluster-Randomized Controlled Trial

**DOI:** 10.3390/nu18213654

**Published:** 2026-11-03

**Authors:** Chaitra A. Kilpady, Akshata A. Kamath, Aswathi Saji, Sridevi Annapurna Singh, Usha Dharmaraj, Chakkaravarthi Arugakeerthy, Vanajakshi Vasudeva, Nirupama Shivakumar, Anura V. Kurpad

**Affiliations:** 1 Centre for Doctoral Studies, Manipal Academy of Higher Education, Manipal 576104, Karnataka, India; chaitra.ak@sjri.res.in (C.A.K.); akshata.ak@sjri.res.in (A.A.K.); aswathi.s@sjri.res.in (A.S.); 2 Division of Nutrition, St. John’s Research Institute, St. John’s National Academy of Health Sciences (A Unit of CBCI Society for Medical Education), Bangalore 560034, Karnataka, India; 3 Division of Epidemiology and Biostatistics, St. John’s Research Institute, St. John’s National Academy of Health Sciences (A Unit of CBCI Society for Medical Education), Bangalore 560034, Karnataka, India; 4 Department of Traditional Foods and Applied Nutrition, CSIR- Central Food Technological Research Institute, Mysuru 570020, Karnataka, India; ssingh98@yahoo.com; 5 Department of Grain Science and Technology, CSIR- Central Food Technological Research Institute, Mysuru 570020, Karnataka, India; usha.cftri@csir.res.in; 6 Department of Food Engineering, CSIR- Central Food Technological Research Institute, Mysuru 570020, Karnataka, India; chakkaravarthi.cftri@csir.res.in; 7 Food Safety & Analytical Quality Control Laboratory, CSIR- Central Food Technological Research Institute, Mysuru 570020, Karnataka, India; vanajakshi.cftri@csir.res.in; 8 Department of Physiology, St. John’s Medical College, St. John’s National Academy of Health Sciences (A Unit of CBCI Society for Medical Education), Bangalore 560034, Karnataka, India; a.kurpad@sjri.res.in

**Keywords:** linear growth, body composition, Take-Home Ration, cluster-randomized controlled trial, nutrition intervention, infants

## Abstract

**Background/Objectives**: Linear growth faltering remains a major public health concern in low- and middle-income countries, wherein sub-optimal complementary feeding and hygiene practices are key modifiable factors. Inadequate complementary feeding is addressed through government-issued Take-Home Ration (THR) for <3-year-old children. This THR is not consumed regularly and does not bridge deficits in protein quality and fat intake among South Indian rural children. The addition of milk and legumes into a reformulated THR would address these gaps, but its efficacy remains unknown. **Methods**: In this 3-month cluster-randomized controlled trial among rural infants (6–9 months old), the intervention group (12 village clusters; *n* = 67) received a modified milk-and legume-based THR along with standard government THR, while the control group (12 village clusters; *n* = 67) received standard government THR alone. Caregivers of both groups were educated on nutrition and hygiene practices. Anthropometry, body composition (BC), morbidity, diet, and compliance data were collected. The primary outcome (length-for-age z-score (LAZ)) and secondary outcomes were analyzed using cluster-adjusted linear mixed models (LMM) in an intention-to-treat analysis. **Results**: While LAZ did not differ significantly between groups (β = 0.063 LAZ/month, 95%CI: −0.019, 0.145; *p* = 0.130), adjusted LMM showed significant differences in length (0.213 cm/month, 95%CI: 0.025, 0.400, *p* = 0.027), weight (0.040 kg/month, 95%CI: 0.001, 0.079, *p* = 0.044) and head circumference (0.067 cm/month, 95%CI: 0.005, 0.128, *p* = 0.033). LAZ decreased in both groups, with a significantly lower decline in the intervention group (slope difference: β = 0.05, 95% CI: 0.006, 0.095, *p* = 0.027). BC did not differ significantly. **Conclusions**: Improving protein quality and fat content of the government-issued THR showed a favourable LAZ trajectory among infants from rural South India. (Clinical Trial Registry-India: CTRI/2020/06/025825).

## 1. Introduction

Child undernutrition has been recognized as a major public health issue, primarily in low- and middle-income countries (LMICs), as it leads to high disease burden and mortality rates, along with impaired cognitive and physical development [1]. Linear growth faltering, a form of chronic undernutrition with multifactorial causation, can particularly heighten the risk of chronic diseases during adulthood and hamper economic productivity [1,2,3]. In the Indian state of Karnataka, the present study location, one-third of children under the age of 5 years were reported to have <−2 length-for-age z-scores (stunting) [4]. Inadequate complementary feeding practices are one of the key factors [5] affecting linear growth faltering. The age of 6 months marks a period of critical nutritional transition, as infants’ increasing energy and nutrient needs can no longer be met by breast milk alone. This makes the introduction of adequate, diverse, and nutrient-dense complementary foods (CF) essential while breastfeeding continues [6]. CF is crucial for the growing infant, with an emphasis on educating mothers on the timing, frequency, quality, quantity, and responsive feeding [6,7].

The Integrated Child Development Services (ICDS) Scheme in India is a visionary system that tries to improve the food intake of young children through a Supplementary Nutrition Programme (SNP). This is offered to children aged 6–36 months through a Take-Home Ration (THR) that allows mothers to feed their children at home. The THR is predominantly cereal-based without any added fat. A situational analysis involving 3-day non-consecutive dietary recalls in ICDS child beneficiaries aged 6–12 months in a rural district of Karnataka identified an energy deficit of ~150 kcals, with 22% fat inadequacy, along with micronutrient inadequacy. This persisted despite breastfeeding, complementary feeding and THR provision and consumption [8]. This highlights the need for a THR with adequate fat and protein content, as well as enhanced energy density (given the low gastric capacity of infants) [5], while also promoting dietary diversity and ensuring its acceptability.

Protein quality is also important, as the Digestible Indispensable Amino Acid Score (DIAAS) of complementary food proteins was inversely correlated with stunting in Indian infants [9]. In consequence, the incorporation of animal protein foods like milk or egg has been recommended to improve the DIAAS of THR protein [9,10]. Animal-source foods provide high-quality protein and essential amino acids that support linear growth and may help reduce growth faltering among young children in LMICs [10]. A systematic review of complementary feeding interventions in developing countries also suggested that education on promoting animal-source food consumption may positively influence growth [11]. A recent evaluation of the THR by the World Food Programme (WFP) recommended improving its composition by increasing fat and micronutrient content and incorporating milk solids to enhance protein quality [12]. However, the impact of reformulating the government THR to address identified nutritional gaps, particularly those related to protein quality and fat content, remains insufficiently evaluated within the SNP.

In addition to anthropometric measurements, the assessment of body composition (BC) during this phase is important [13]. Studies have reported mixed effects of high protein and fat intake on Fat-free Mass (FFM) and Fat Mass (FM) in children [14,15,16]. Further, educating mothers on Infant and Young Child Feeding practices (IYCF) and Water, Sanitation and Hygiene (WASH) has also been shown to have a positive impact on child linear growth [17]. However, there are limited studies examining responses to an integrated approach to maintaining linear growth and FFM in Indian settings, especially among ICDS beneficiaries [18,19].

Therefore, the present study aimed to test the impact of a 3-month-long intervention with a modified milk- and legume-based THR (ML-THR), formulated to address the observed nutrient gap [20], on the linear growth (primary outcome) and BC of infants aged 6–9 months in comparison to those receiving the standard government THR (SG-THR) alone. The study hypothesized primarily that the ML-THR would reduce the growth faltering that is typically observed in such children [4] in comparison to those receiving only SG-THR, and secondarily, that a relatively higher FFM would result in the ML-THR group.

## 2. Materials and Methods

### 2.1. Study Design and Participants

The present study was a community-based cluster-randomized controlled trial conducted in two rural sub-districts, Hiriyur and Molkalmuru, of Chitradurga district, in the Indian state of Karnataka. In this district, an earlier situational analysis had shown that deficits in nutrient intakes were high [8], and the sub-districts were randomly selected from a total of seven sub-districts for this study. The participants were infants registered as beneficiaries under the ICDS Scheme. Apparently healthy infants aged 6–9 months who were either exclusively or primarily breastfed were recruited, as this is the age group where complementary feeding is initiated, and THR is first received. Breastfeeding status at enrolment was determined through maternal self-report during screening and was subsequently reconfirmed during the baseline assessment. Infants diagnosed with chronic medical or surgical conditions that impair growth, congenital syndromes, severe acute malnutrition (weight-for-length z-scores < −3), or a maternal-reported history of allergy to milk or legumes were excluded.

Infants were screened for inclusion and exclusion criteria. Written informed consent was obtained from primary caregivers. The study received ethical approval from the Institutional Ethics Committee, St. John’s Medical College and Hospital, Bengaluru, prior to study commencement (IEC Reference No: 83/2019, 12 April 2019), supported by the state’s Department of Health and Family Welfare and Department of Women and Child Development. Among consented participants, developmental history was obtained to assess the attainment of age-appropriate developmental milestones. A physical examination was also performed by pediatricians to identify any abnormalities or clinical conditions that could potentially affect the child’s growth or development. The trial was prospectively registered with the Clinical Trial Registry-India [Registration number: CTRI/2020/06/025825, registered on 11 June 2020 (available at: https://ctri.nic.in/Clinicaltrials/pubview.php) (accessed on 27 August 2026).

### 2.2. Sample Size, Randomization and Intervention

The sample size was based on a previous study in Bangladesh which showed a 0.19 (95% CI: 0.12, 0.27) change in LAZ in 3 months in response to milk, egg, and multiple micronutrient supplementation [21]. Considering the upper confidence limit as the expected difference (0.27 units) with an assumed SD of 0.4, 80% power and a 5% level of significance, the sample size required was 35 per group, which was rounded off to 40 in the control and intervention groups. A cluster-randomized controlled trial conducted earlier reported an intra-cluster correlation coefficient of 0.129 for LAZ [22]. Expecting a similar intra-cluster correlation coefficient, the design effect was 1.52, considering 5 eligible infants per cluster. The resulting cluster-adjusted sample size was ~61 participants per group. An additional 10% was added to account for dropout, resulting in 67 participants per group. The ICDS program operates within village clusters, with a minimum of 20 creches per cluster. For the study, 12 village clusters were randomly selected per group, yielding a 1:1 allocation ratio. Cluster randomization was followed at the village cluster level (Supplementary Figure S1). Randomization was performed using a computer-generated sequence by a research staff member. Blinding of the research staff and participants was not feasible during data collection due to logistical constraints; however, the data analyst was blinded.

The duration of the ML-THR intervention was 3 months. Participant recruitment commenced in 2022 and continued through 2023. Both groups were educated on IYCF and WASH, while they continued to receive the SG-THR from the ICDS program. The SG-THR, which was earlier determined to have a low compliance rate [8], consisted of a monthly pack with raw ingredients, consisting of 2 kg broken rice, 570 g whole mung bean, 350 g split mung bean, 1450 g jaggery (raw sugar), and 300 g whole milk powder, targeting a daily calorie and protein recommendation of 500 kcal and 12–15 g, respectively. The ML-THR was formulated to meet the previously observed nutrient gap in this district, using local ingredients, within the cost constraint of the Indian Government mandate (INR 8/day), with an energy content of ~200 kcal, energy density of ~1.5 kcal per gram (cooked product) and a protein-energy ratio of 12% [20]. This was tested extensively for shelf life and safety. Further details on shelf-life testing have been mentioned in Supplementary Material S1, and its nutrient composition is given in Supplementary Table S1.

The ML-THR was produced on a biweekly basis at the CSIR-Central Food Technological Research Institute (CFTRI), Mysuru, India, with microbiological analysis for every batch, adhering to the Bureau of Indian Standards [23]. The shelf life of the product was tested under two conditions: ambient (27 °C, 64% relative humidity) for 120 days and accelerated (38 °C, 92% relative humidity) for 90 days, and a shelf life of 4 months was reported (Supplementary Material S1). The ML-THR was delivered biweekly to the participants in the form of packs (322 g, with 7 single-day packs of 46 g) for daily consumption, and a demonstration on cooking the product was carried out by the field staff.

ML-THR compliance checks were conducted biweekly. The intervention period was calculated from the day of ML-THR distribution to the endline data collection. The total amount consumed was calculated as the amount of ML-THR per packet (46 g) multiplied by the number of intervention days. Empty, partially used and unused sachets were weighed by the study team. Sachets that were not returned were considered as unused in the compliance calculation. The total grams consumed (actual intake) and compliance were calculated as follows:

Expected intake = 46 g × no. of intervention days

Actual intake = (46 g × intervention days) − Remaining amount in the packets collected

Compliance % = (Actual intake/Expected intake) × 100

### 2.3. IYCF and WASH Education

Education materials (booklet and animated videos) were developed to impart knowledge to the caregivers, incorporating concepts of IYCF and WASH practices recommended by the World Health Organization (WHO) [24] and United Nations Children’s Fund (UNICEF) [25]. The booklet also consisted of different recipes that met the energy, protein and fat requirements of infants based on the WHO complementary food guidelines [24]. The content was tailored for the target audience in their local dialect. At baseline, the contents of the booklet were explained by nutritionists to caregivers of both groups. The topics were reinforced and revisited as appropriate during every house visit by the trained field staff. Knowledge of caregivers was assessed using pre- and post-education questionnaires. To promote hygiene, buckets and soap were also provided to both groups.

### 2.4. Data Collection, Questionnaires, Anthropometry and Body Composition Measurements

Questionnaires were administered by trained field staff to acquire data on demographics and socio-economic status [26] and the Household Food Insecurity Access Scale [27]. At each follow-up visit, morbidity during the preceding 30 days was collected through maternal recall. Morbidity episodes were recorded based on reported symptoms or diagnoses that occurred during this period, including diarrhea (≥3 loose/liquid stools per day), abdominal pain, vomiting, cough, runny nose, sore throat, fever (axillary temperature ≥ 37.5 °C), breathing difficulty, ear pain, and hospitalization. At baseline and endline, complete blood count, including hemoglobin (ABX-Pentra 60C+, Horiba Medical, Kyoto, Japan), was measured, and 24 h dietary recalls for 3 non-consecutive days (2 weekdays and 1 weekend) were collected using a standardized portion kit. The nutrients from the recall were calculated using the NSR-Nutrical software [28].

Monthly anthropometric measurements by trained field staff included length using a standard infantometer (Seca 417; Seca GmbH & Co. KG, Hamburg, Germany) to the nearest 0.1 cm. Weight was measured by a standard calibrated Seca 874 digital flat scale (Seca GmbH & Co. KG, Hamburg, Germany) to the nearest 10 g. Head circumference and mid-upper arm circumference (MUAC) were measured using a standard non-flexible measuring tape (Seca 212; Seca GmbH & Co. KG, Hamburg, Germany). Measurement values were double-entered and transformed to z-scores using WHO Anthropometry software (version 3.3.2; World Health Organization, Geneva, Switzerland). Infants with z-scores < −2 SD for length-for-age (LAZ), weight-for-length (WLZ), and weight-for-age (WAZ) were classified as stunted, wasted, and underweight, respectively. The technical errors of measurement (TEM) and relative TEM (%) for anthropometric measurements were calculated among three trained field staff at three time points (baseline, midline, and endline). Intra-observer and inter-observer variability were found to be below their respective acceptable cutoffs of 1.0% and 1.5% [15,29].

Body composition was measured at baseline and endline using the deuterium dilution technique as per the International Atomic Energy Agency (IAEA) protocol [30]. About 1 mL of basal saliva sample was collected using sterile cotton prior to dosing. The weight of the child was measured, and a deuterium oxide (D2O; 99.8 atom % 2H) dose of 0.05 g/kg body weight [31] was accurately weighed and administered. The protocol was terminated in case of any dose spillage/regurgitation. The participant was allowed to drink water/breastfeed or consume food 1 h after dosing, noting the time. About 1 mL of saliva sample was collected at 3 and 3.5 h after dose. Saliva was collected at least 15 to 20 min after feeding to ensure there was no food or milk in the mouth [32]. A pre-weighed water sipper was given to account for the volume consumed. The saliva sample was aliquoted into two airtight sterile cryovials with a volume of ~0.5 mL and stored at −20 °C [33]. These were later analyzed in duplicate using Fourier-transform infrared spectrometry technique (FTIR, Agilent 4500 Series, Santa Clara, CA, USA) at St. John’s Research Institute, Bengaluru [33]. The 3rd hour values were considered for estimating body composition, supported by a previous study [32]. Total Body Water (TBW) was determined from the ratio of the administered dose of deuterium oxide and the deuterium enrichment of body water, adjusted for non-aqueous hydrogen exchange [30]. FFM was calculated from TBW using age- and sex-specific hydration values [33] and FM was computed as the difference in body weight and FFM.

### 2.5. Statistical Analysis

All inferential analyses were based on the intention-to-treat principle. Data normality was assessed using Q-Q plots and the Shapiro–Wilk test. Continuous variables were presented as mean ± standard deviation or median (Q1, Q3) and categorical variables as frequencies and percentages. Analyses were performed using available observations, with missing data handled without imputation. Between-group comparisons for anthropometric and BC parameters were done using Student’s *t*-test or Mann–Whitney *U* test, with a similar statistical approach for sex-stratified comparisons, considering prior evidence of sex-specific differences [15].

Primary analysis: As the study was powered to detect a difference in LAZ between baseline and endline, the primary analysis was restricted to these two timepoints. The intervention effect on LAZ (primary outcome) was estimated using a linear mixed model (LMM) with group, time and the group × time interaction as fixed effects, and random intercepts for village clusters and for participants nested within clusters, to account for the cluster-randomized design and repeated measurements.

More specifically, outcomes were analyzed using three-level linear mixed models, with repeated measurements (level 1) nested within infants (level 2), which are nested within village clusters (level 3). The model was:

*Y_ijk_* = *β*_0_ + *β*_1_*Group_k_* + *β*_3_(*Group_k_* × *Time_ijk_*) + *γ^T^X_jk_* + *u_k_* + *v_jk_* + *ε_ijk_*

$$u_{k}\sim N(0,\sigma_{u}^{2}),\;v_{\mathrm{jk}}\sim N(0,\sigma_{v}^{2}),\varepsilon_{\mathrm{ijk}}\sim N(0,\;\sigma_{\epsilon }^{2})$$

*Y_ijk_* is the outcome measured at measurement time points (baseline/endline/monthly measurements) *i* for infant *j* in village cluster *k*. Time represents the study visit; therefore, the group × time interaction coefficient (*β*_3_) represents the between-group difference in the rate of change per month. *X_jk_* denotes the covariates, while *u_k_*, *v_jk_* are random intercepts which account for village clustering and for participants within clusters. *ε_ijk_* is the residual error. Models were fitted with and without adjustment for sex, age, morbidity and post-education knowledge score; the adjusted estimate was considered the primary result. Details of the covariates have been given in the subsequent sections below.

Secondary analyses: Adjusted LMMs were also used to estimate intervention effects on secondary outcomes including length, weight, HC, other anthropometric z-scores, and BC measures. These models were adjusted for the same covariates as the primary model. To assess the association between ML-THR adherence (percentage compliance) and outcome measures, LMMs were fitted in the intervention group alone, adjusting for the same covariates and percentage compliance.

Supportive analyses: Linear growth trajectory was assessed using a group × time interaction model to evaluate differences in the rate of change in LAZ across the 4 time points (baseline, 1st month, 2nd month, and endline). Additionally, the velocity (calculated as the 3-month change) of linear growth and weight, and change in FFM%, were compared between groups using an independent *t*-test. An unadjusted (without accounting for cluster design) difference-in-difference (DID) analysis was also performed to estimate the effect of the intervention on anthropometric outcomes, z-scores, and BC measures as part of exploratory analyses.

In morbidity, the prevalence of illnesses was compared between groups using chi-square or Fisher’s exact test, as appropriate. Illness episode counts were generated for each participant-month by summing up the number of reported illnesses (for overall morbidity) and diarrheal episodes. To account for repeated observations within participants and village cluster-level variation, Poisson mixed-effects regression models were fitted to examine incidence rate ratios (IRRs) for overall morbidity (all reported illnesses) and diarrhea-specific morbidity.

The usual nutrient intake distribution was obtained from the 24 h dietary recalls (Supplementary Material S2). Comparisons between groups at both time points were done using Student’s *t*-test or the Mann–Whitney *U* test. The minimum dietary diversity (MDD) and dietary diversity score (DDS) were derived from the 24 h dietary recall and calculated according to the WHO/UNICEF guidelines [34]. Fisher’s exact test or the chi-square test was used for between-group comparisons.

For IYCF and WASH education, caregiver responses were converted to percentages. Knowledge scores were classified as: Poor (0–50%), Fair (51–80%) and Good (81- 100%) [35]. Chi-square test or Fisher’s exact test were used to compare knowledge categories between groups at both time points, and McNemar’s test was used for within-group changes.

The data analyst was blinded to the group allocation to minimize potential analytical bias. Data analysis was conducted using R version 4.5.2. A *p*-value < 0.05 was considered statistically significant.

## 3. Results

### 3.1. Recruitment and Baseline Characteristics

**Figure 1:**
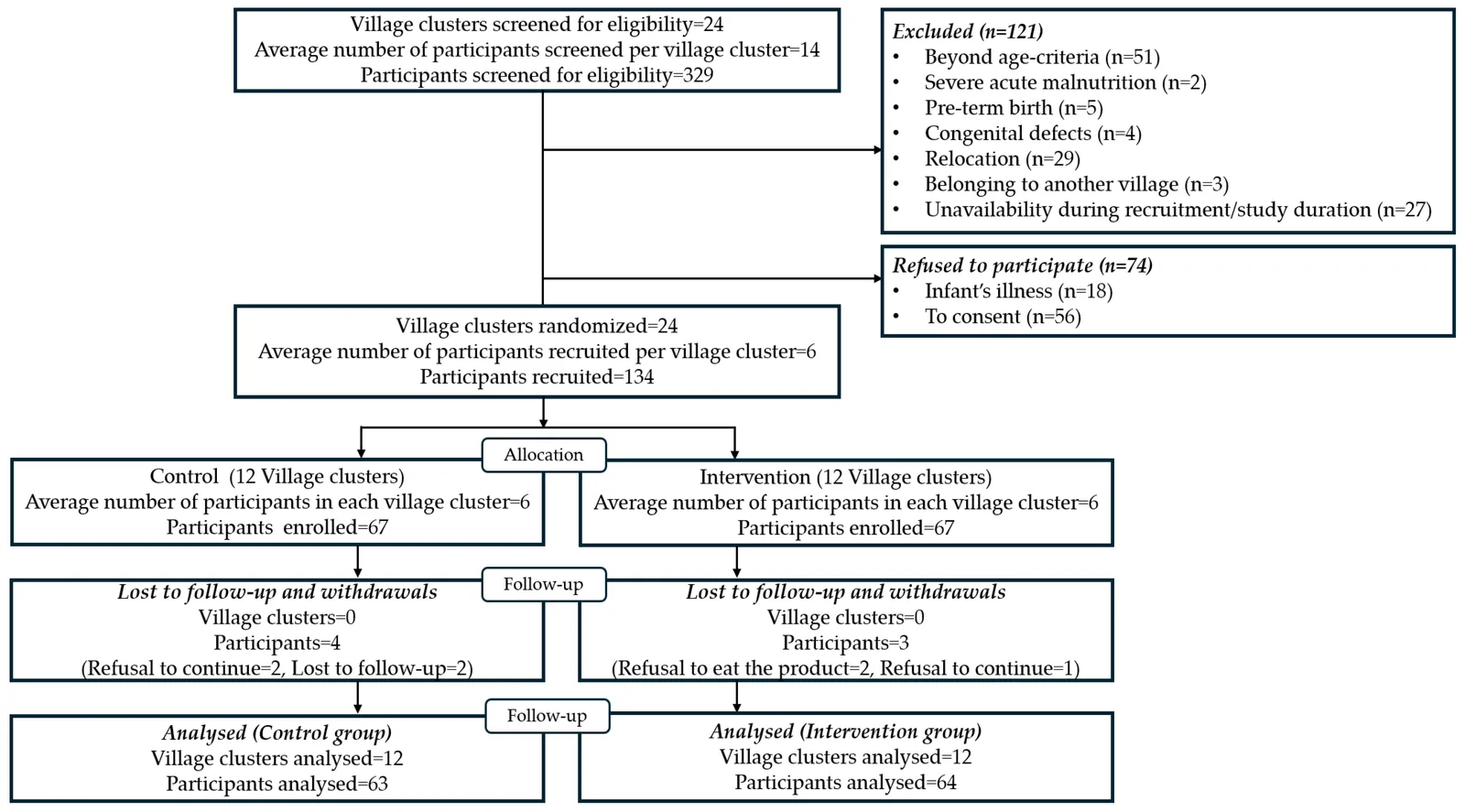
Screening and recruitment CONSORT diagram.

A total of 134 infants were recruited and equally allocated to the control group (12 village clusters) and the intervention group (12 village clusters). The flow of clusters and participants through the trial is shown in Figure 1. Baseline characteristics of the participants were comparable between the two groups across all study variables (Table 1).

### 3.2. Anthropometry and Body Composition Measures

**Figure 2:**
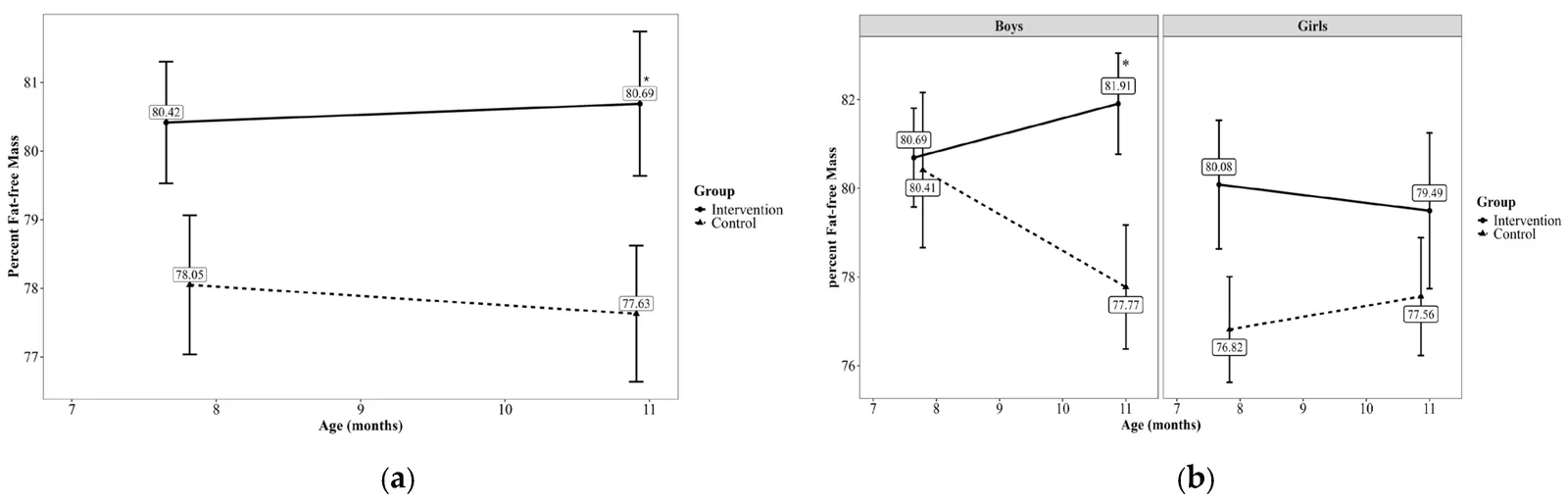
(**a**) Fat-free mass % with age; (**b**) Fat-free mass % with age and sex. * indicates statistical significance at *p* < 0.05.

The monthly anthropometric measures along with z-scores, baseline and endline BC, and respective sex-wise measurements of participants have been provided in Supplementary Tables S2–S4. At endline, a relatively lower prevalence of stunting (28.1% versus 32.3%), wasting (4.7% versus 6.5%), and underweight (21.9% versus 23.8%) was noted in the intervention compared to the control group, but was not statistically significant (Supplementary Table S2). In BC, the intervention group showed an increase in FFM% compared to controls (80.7% versus 77.6%, *p* = 0.037) at endline (Figure 2a). A similar pattern was observed among boys of the intervention group (Figure 2b).

### 3.3. Effect of Intervention on Anthropometry and Body Composition

In the adjusted LMM, the between-group difference in the rate of change in LAZ was not statistically significant (β = 0.063 LAZ/month, 95% CI: −0.019, 0.145; *p* = 0.130), Table 2. Among secondary outcomes, the intervention group showed greater monthly gains in length (β = 0.213 cm/month, 95% CI: 0.025, 0.400; *p* = 0.027), weight (β = 0.040 kg/month, 95% CI: 0.001, 0.079; *p* = 0.044) and head circumference (β = 0.067 cm/month, 95% CI: 0.005, 0.128; *p* = 0.033). No between-group differences were observed for WAZ, WLZ, HCZ or body composition measures (Table 2).

BC was assessed as a secondary outcome. Usable samples were available for 35 control and 43 intervention participants at baseline, and 28 control and 40 intervention participants at endline, with paired baseline and endline samples available for 25 control and 37 intervention participants. Samples were unavailable due to non-collection, low enrichment, insufficient sample volume, dose spitting, refusal to provide post-dose saliva and loss to follow-up. However, baseline characteristics were comparable between participants whose BC was measured and not measured (Supplementary Table S5).

The unadjusted DID estimates of the total change over the intervention period were consistent in direction and magnitude (Supplementary Table S6).

The mean compliance of ML-THR was 79.9 ± 17.3% during the intervention period (mean of 92 ± 1 days). No allergy related to the product was reported during the study period. In secondary analysis within the intervention group, % ML-THR compliance was significantly associated with head circumference (β = 0.02 cm/month, 95% CI: 0.00, 0.04; *p* = 0.046) but not with other anthropometric or BC measures (Table 3).

**Figure 3:**
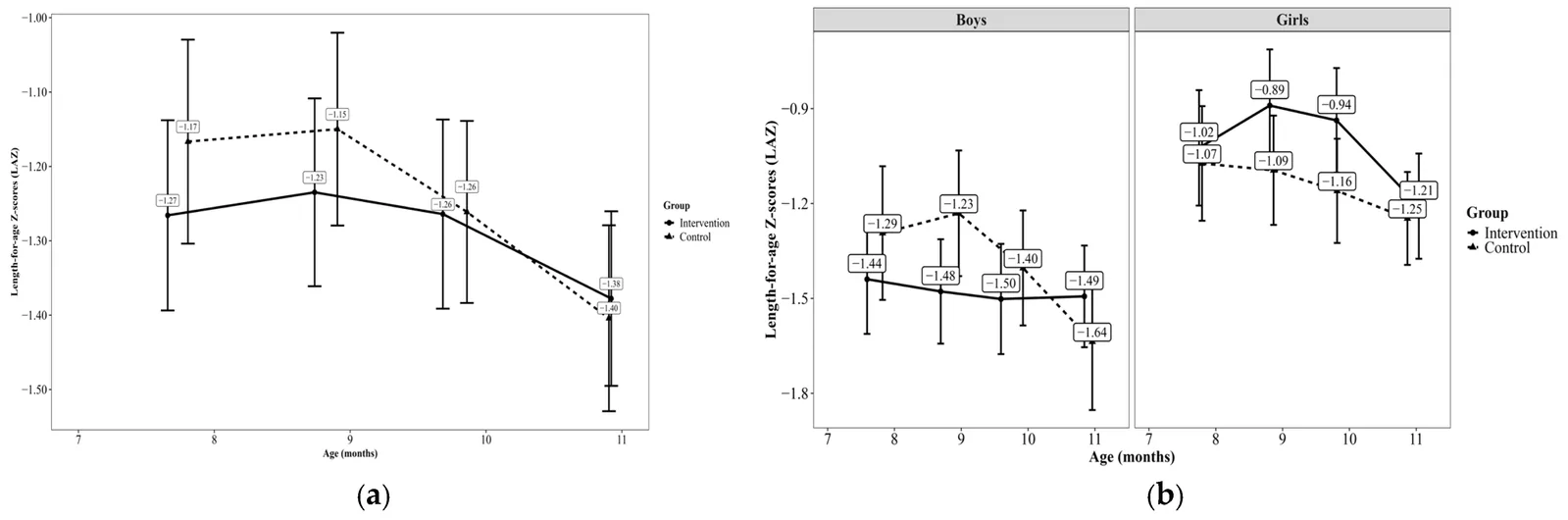
(**a**) Length-for-age z-scores with age; (**b**) Length-for-age z-scores with age and sex.

As part of exploratory analyses, a decline in LAZ was observed in both groups over time (considering all 4 timepoints-baseline, 1st month, 2nd month and endline). However, the control group (β = −0.066, 95% CI: −0.098, −0.034) showed a steeper decline in LAZ when compared to the intervention group (β = −0.016, 95% CI: −0.047, 0.016) with a difference in slope of β = 0.05, 95% CI: 0.006, 0.095; *p* = 0.027, Figure 3a. There were no significant differences observed between groups across time points for WAZ and WLZ trajectories (Supplementary Figures S2a and S3a, respectively). The overall mean linear growth or weight velocity was similar (1.19 versus 1.06 cm/month, *p* = 0.123 and 213 versus 217 g/month, *p* = 0.907) between the intervention and control groups, respectively. The change in FFM% was 0.79 (95% CI: −0.43, 2.02) in the control group and 0.83 (95% CI: −1.02, 2.68) in the intervention group (*p* = 0.970).

### 3.4. Morbidity

The prevalence of individual illnesses did not differ between the control and intervention groups across time points (Supplementary Table S7). However, at endline there was a higher prevalence of diarrhea in the control group (control: 30.6% versus intervention: 14.3%; *p* = 0.048). The adjusted incidence rate ratio of ‘overall morbidity’ and ‘diarrhea’ indicated no differences between groups (Table 4). Infants in both groups experiencing morbidity were treated by the local pediatricians as per standard clinical practice.

### 3.5. Nutrient Intake

The usual intake of nutrients among the groups was similar at baseline. Higher intakes of energy, protein, fat, carbohydrates, calcium, phosphorus, iron, zinc, vitamin A, vitamin B6 and vitamin B12 were observed in the intervention group (*p* < 0.05) when compared to the control group at endline (Table 5). Similar patterns were observed in usual intakes of legumes, nuts and milk products, which could be attributed to the intervention (Supplementary Table S8). MDD and DDS derived from 24 h dietary recall data were comparable between groups at baseline. At endline, the intervention group showed higher MDD and DDS than the control group (MDD: 84.1% versus 60.7% and DDS: 5.3 ± 1.0 versus 4.8 ± 1.4; *p* < 0.01 for both; Table 6).

### 3.6. Pre- and Post-Education Scores on IYCF and WASH Domains

A significant improvement was observed in mean scores following the caregiver education within groups for domains such as IYCF, WASH, and combined education (Supplementary Table S9). At baseline, the knowledge (IYCF and WASH combined) of caregivers of both groups was distributed across 3 categories—“poor” (control: 3.0% and intervention: 1.5%), “fair” (control: 66.7% and intervention: 68.2%) and “good” (control: 30.3% and intervention: 30.3%). Post-education, no caregivers remained in the “poor” knowledge category. Only a small proportion (control: 6.4% and intervention: 6.5%) were in the “fair” knowledge category, with a significant increase in scores (*p* < 0.001), showing a majority in the “good” knowledge category in both groups (control: 93.6% and intervention: 93.7%) (Supplementary Table S10). There were no differences observed between groups at both time points.

## 4. Discussion

The present cluster-randomized controlled trial evaluated the effect of a 3-month intervention with a modified milk-legume-based Take-Home Ration (ML-THR), which was formulated to bridge a previously observed nutrient gap [8], on linear growth and body composition in infants aged 6–9 months, belonging to rural sub-districts of Karnataka, South India. IYCF and WASH education was provided to both the study groups. While a significant group × time interaction for LAZ suggested a more favourable trajectory in the intervention group over the study period, as hypothesized, and was associated with gains in absolute anthropometric measures, this did not translate into a significant adjusted LMM estimate in LAZ (β = 0.063 LAZ/month, 95% CI: −0.019, 0.145; *p* = 0.130), which reflects growth relative to age-sex-specific reference standards. The smaller decline in LAZ in the intervention group suggests a potentially favourable trend, although the findings do not establish a significant intervention effect on the standardized growth over the 3-month period. The mean length velocity in the intervention group (1.19 versus 1.06 cm/month in controls) nearly matched the WHO (~1.3 cm/month) standards [36]. Previously, a study with a comprehensive intervention package (egg- milk based snack, multiple nutrient powder and counselling on child feeding and handwashing) for 12 months in Bangladeshi infants (average age ~ 9 months) showed a similar rate of length gain (1.20 versus 1.04 cm/month) in the intervention compared to the control group [37]. Another longitudinal study evaluating growth trajectories in urban South Indian infants born appropriate for gestational age reported a similar length gain of 1.2 cm/month between 9 and 12 months [38]. Complementary feeding may influence growth through both the nutritional quality of foods offered and feeding practices. A randomized trial in Brazil [39] reported differences in BMI-for-age z-scores across complementary feeding approaches, but no significant differences in LAZ. In contrast, an RCT in breastfed children in India found that supplementation with milk-cereal mix improved LAZ in the high-protein group (adjusted mean difference 0.08; 95% CI 0.01, 0.15 [19]. These studies suggest that nutritional quality and feeding practices may influence growth differently. A systematic review of complementary feeding interventions in LMICs highlighted that interventions emphasizing animal-source foods were more likely to benefit growth [11], consistent with the milk-based component evaluated in the present study.

Our findings are consistent with evidence from short-term nutritional intervention studies in similar settings. Malawian infants (9–12 months) who received bovine colostrum/egg powder for 3 months showed that, although growth faltering occurred in both groups, it was less in the intervention group (N = 137), with a significantly higher delta LAZ score (0.12, *p* = 0.0011) [40]. Another study in stunted and at-risk of stunting Bangladeshi infants (N = 472, 12–18 months) showed that a 3-month intervention with milk, egg and micronutrient powder significantly (*p* < 0.001) increased LAZ by 0.23 units [21]. These studies support incorporation of animal source proteins in complementary feeds to promote linear growth [10]. The lack of significant improvement in LAZ in our study, compared to these trials, may be explained by differences in baseline nutritional status, as well as variations in type and duration of intervention, sample size and statistical power. In support, a study with a similar sample size, conducted in infants (4–6 months) at risk of stunting, belonging to slums in Bangladesh, showed that the group (N = 43) receiving yogurt plus nutrition education had a greater but insignificant change in linear growth (LAZ: mean difference 0.20, 95% CI: −0.06, 0.47; *p*-value = 0.13) compared to controls. Other potential explanations for the modest change in LAZ include environmental enteropathy and the multifactorial determinants of child growth [41]. In our study, key influencing factors such as morbidity and IYCF/WASH knowledge were comparable between groups. Although IYCF/WASH education was provided to both groups and caregiver knowledge improved comparably, this common component may have improved feeding-related behaviours in both groups and may have potentially reduced the differences attributed specifically to ML-THR.

The intervention group demonstrated a higher FFM% at endline; however, the adjusted LMM results were statistically insignificant. Evidence on infant BC responses to nutritional interventions using robust methods such as deuterium dilution, dual-energy X-ray absorptiometry (DEXA), or air displacement plethysmography (ADP) remains limited due to their technical complexity. Existing trials suggest that energy- and nutrient-dense supplementation can increase both FFM and FM, with some evidence of sex-specific responses [15]. Notably, gains in FFM appear to be more responsive to such interventions than FM, although findings are not entirely consistent across settings and populations [15,42]. A lipid-based nutrient supplement trial in Burkina Faso among children aged 6–23 months with moderate acute malnutrition demonstrated a significantly higher FFMI by 0.083 kg/m^2^ (95% CI 0.003, 0.183; *p* = 0.042), using the deuterium dilution technique, compared to a corn-soy blend control [42]. Similarly, an RCT of four complementary food supplements provided over one year from 6 months of age reported higher total FFM(g) and FM(g) among children receiving Plumpy’doz and chickpea-based foods relative to controls. Sex-specific analyses indicated differential responses, with girls showing greater gains in FFM(g) and FM(g) with Plumpy’doz compared to those in the control group [15].

A key strength of this study was its targeted approach to addressing the identified nutritional gap through a modified THR that was acceptable among infants, while testing its effects on growth. The application of the deuterium dilution technique to assess BC in a community-based setting further enabled the evaluation of changes beyond anthropometric measures. Additionally, the integrated design, incorporating dietary assessment, morbidity, and IYCF/WASH education, enabled a comprehensive evaluation of growth determinants. A major limitation was the short intervention duration due to financial constraints and a small sample size. Body composition analyses were conducted in a smaller subset of participants due to limited data availability, highlighting the need for larger studies to obtain more precise estimates and further elucidate between-group differences in body composition outcomes. However, in the present study, baseline characteristics were comparable between participants with and without available data. Compliance was monitored based on leftover packet quantity and not through direct observation. Another limitation was the inability to implement blinding, as the SG-THR received from the Government consisted of raw ingredients, whereas the ML-THR was provided in powdered form. However, the individual responsible for data analysis was blinded to group allocations to minimize potential analytical bias. The dietary intake and morbidity data were based on caregiver reports and may be subject to recall or reporting bias. Because both groups received IYCF/WASH education, its independent contribution to the observed outcomes could not be determined. The findings provide insights into the intervention’s effects in rural Karnataka, highlighting the need for validation in diverse populations.

## 5. Conclusions

In conclusion, this 3-month intervention with ML-THR showed a positive trend toward improved LAZ. Longer-duration studies are recommended to understand the sustained impacts on linear growth.

## Figures and Tables

**Table 1:** Baseline characteristics of study participants.

Variables	Control	Intervention
***N***	66	67
**Boys, *n* (%)**	28 (42.4)	37 (55.2)
**Site (Sub-district), *n* (%)**		
Site 1 (Hiriyur)	45 (68.2)	48 (71.6)
Site 2 (Molkalmuru)	21 (31.8)	19 (28.4)
Age (months) *	7.79 ± 0.57	7.72 ± 0.65
Birthweight (kg) *	2.78 ± 0.51	2.85 ± 0.45
**Birth order of the participant, *n* (%)**		
1	33 (50.0)	33 (49.3)
2	27 (40.9)	21 (31.3)
>=3	6 (9.1)	13 (19.4)
**Mother’s education, *n* (%)**		
Illiterate	8 (12.1)	8 (11.9)
Primary School (1st–5th std)	3 (4.5)	6 (9.0)
Middle School (6th–8th std)	7 (10.6)	4 (6.0)
High School (9th–10th std)	23 (34.8)	16 (23.9)
PUC/Diploma	23 (34.8)	24 (35.8)
>=Graduation	2 (3.0)	9 (13.4)
Monthly Income (INR) *	10,939.39 ± 5900.79	10,791.04 ± 8126.91
**Socioeconomic class, *n* (%)**		
Upper	12 (18.2)	12 (17.9)
Upper middle	2 (3.0)	4 (6.0)
Lower middle	19 (28.8)	22 (32.8)
Upper lower	32 (48.5)	28 (41.8)
Lower	1 (1.5)	1 (1.5)
**Household food security status, *n* (%)**		
Secure	55 (83.3)	61 (91.0)
Mildly insecure	5 (7.6)	0 (0.0)
Moderately insecure	1 (1.5)	3 (4.5)
Severely insecure	5 (7.6)	3 (4.5)
**No. of breastfed infants, *n* (%)**	66 (100.0)	67 (100.0)
Initiation of complementary foods (age in months) *	6.45 ± 1.08	6.67 ± 0.89
Quantity of THR received *		
Pushti (kg)	2.95 ± 0.22	2.94 ± 0.32
Jaggery (kg)	1.13 ± 0.31	1.19 ± 0.24
Whole milk powder (g)	330.95 ± 134.83	297.66 ± 108.54
Broken rice (kg)	2.07 ± 0.05	2.10 ± 0.00
Whole mung bean (g)	468.67 ± 158.76	373.00 ± 0.00
Split mung bean (g)	576.40 ± 97.05	533.00 ± 0.00
**Caregiver reported infant SG-THR use, *n* (%)**	48 (72.7)	55 (82.1)
**Consumption of SG-THR, *n* (%)**		
Only by infant	30 (45.4)	28 (41.8)
Shared among siblings	0 (0.0)	1 (1.5)
Shared among family members	18 (27.3)	26 (38.8)
Anthropometric and Body composition measurements *		
Length-for-age z-scores (LAZ)	−1.17 ± 1.11	−1.27 ± 1.05
Weight-for-age z-scores (WAZ)	−1.17 ± 0.93	−1.07 ± 0.97
Weight-for-length z-scores (WLZ)	−0.59 ± 1.06	−0.40 ± 1.15
Head circumference z-scores	−1.29 ± 0.99	−1.23 ± 0.94
Mid-upper arm circumference z-scores	−0.74 ± 0.85	−0.57 ± 0.89
Stunting (LAZ < −2), *n* (%)	13 (19.7)	13 (19.4)
Wasting (WLZ < −2), *n* (%)	4 (6.2)	3 (4.5)
Underweight (WAZ < −2), *n* (%)	13 (19.7)	8 (11.9)
Overweight (WLZ > −2), *n* (%)	0 (0.0)	2 (3.0)
*N*	35	42
FFM (kg)	5.61 ± 0.60	5.83 ± 0.56
FM (kg)	1.61 ± 0.55	1.44 ± 0.50
FFM %	78.05 ± 5.99	80.42 ± 5.74
FM %	21.95 ± 5.99	19.58 ± 5.74
FFMI (kg/m^2^)	12.63 ± 0.96	13.03 ± 1.12
FMI (kg/m^2^)	3.65 ± 1.23	3.22 ± 1.13
***Blood parameter*** *		
*N*	45	52
Haemoglobin (g/dl)	10.1 ± 1.2	10.3 ± 1.2

Abbreviations: *N*—number of participants, PUC—Pre-university college, SG-THR—Standard Government Take-Home Ration, FFM—Fat-free mass, FM—Fat mass, FFMI—Fat-free mass index, FMI—Fat mass index; Values are expressed as * mean ± SD.

**Table 2:** Linear Mixed Model of anthropometric and body composition measurements.

Variable of Interest	Unadjusted β (95% CI)	*p*-Value	Adjusted * β (95% CI)	*p*-Value	Intra-Cluster Correlation for Participant (Adjusted Model)	Intra-Cluster Correlation for Village Clusters (Adjusted Model)
**LAZ**	0.063 (−0.018, 0.144)	0.127	0.063 (−0.019, 0.145)	0.130	0.721	0.079
**WAZ**	0.026 (−0.018, 0.071)	0.244	0.020 (−0.023, 0.064)	0.359	0.865	0.070
**WLZ**	−0.013 (−0.099, 0.073)	0.769	−0.021 (−0.107, 0.064)	0.622	0.734	0.063
**HCZ**	0.038 (−0.013, 0.089)	0.143	0.040 (−0.012, 0.091)	0.129	0.896	0.000
**Length (cm)**	0.212 (0.026, 0.398)	0.026	0.213 (0.025, 0.400)	0.027	0.757	0.057
**Weight (kg)**	0.046 (0.005, 0.086)	0.026	0.040 (0.001, 0.079)	0.044	0.873	0.060
**HC (cm)**	0.064 (0.003, 0.125)	0.039	0.067 (0.005, 0.128)	0.033	0.913	0.000
**FFM (kg)**	0.119 (−0.057, 0.294)	0.180	0.121 (−0.054, 0.296)	0.173	0.644	0.153
**FM (kg)**	−0.011 (−0.230, 0.208)	0.920	−0.010 (−0.228, 0.209)	0.929	0.727	0.000
**FFM %**	0.385 (−2.180, 2.950)	0.765	0.400 (−2.160, 2.960)	0.755	0.634	0.029
**FM %**	−0.385 (−2.950, 2.180)	0.765	−0.400 (−2.960, 2.160)	0.755	0.634	0.029
**FFMI (kg/m^2^)**	−0.102 (−0.554, 0.350)	0.654	−0.106 (−0.556, 0.344)	0.640	0.480	0.116
**FMI (kg/m^2^)**	−0.100 (−0.547, 0.347)	0.656	−0.103 (−0.549, 0.343)	0.646	0.748	0.000

Note: Abbreviations: LAZ—Length-for-age z-scores, WAZ—Weight-for-age z-scores, WLZ—Weight-for-length z-scores, HC—Head Circumference, FFM—Fat-free mass, FM—Fat mass, FFMI—Fat-free mass index, FMI—Fat mass index; * Adjusted for sex, age, post-education knowledge score, morbidity. Unadjusted and adjusted estimates are reported relative to Control (reference).

**Table 3:** Linear Mixed Model-Intervention percentage compliance with anthropometric and body composition measurements.

Variable of Interest	Adjusted * β (95% CI)	*p*-Value	Intra-Cluster Correlation for Participant (Adjusted Model)	Intra-Cluster Correlation for Village Clusters (Adjusted Model)
**LAZ**	0.009 (−0.006, 0.024)	0.241	0.667	0.148
**WAZ**	0.011 (−0.005, 0.028)	0.159	0.852	0.093
**WLZ**	0.008 (−0.010, 0.026)	0.350	0.811	0.000
**HCZ**	0.015 (−0.001, 0.030)	0.062	0.919	0.000
**Length (cm)**	0.023 (−0.012, 0.059)	0.194	0.000	0.053
**Weight (kg)**	0.012 (−0.003, 0.026)	0.111	0.596	0.072
**HC (cm)**	0.020 (0.000, 0.040)	0.046	0.516	0.000
**FFM (kg)**	0.002 (−0.007, 0.011)	0.709	0.042	0.032
**FM (kg)**	0.004 (−0.008, 0.015)	0.517	0.623	0.000
**FFM %**	−0.035 (−0.155, 0.085)	0.554	0.546	0.000
**FM %**	0.035 (−0.085, 0.155)	0.554	0.546	0.000
**FFMI (kg/m^2^)**	−0.007 (−0.026, 0.011)	0.438	0.498	0.003
**FMI (kg/m^2^)**	0.005 (−0.020, 0.029)	0.699	0.655	0.000

Note: Abbreviations: LAZ—Length-for-age z-scores, WAZ—Weight-for-age z-scores, WLZ—Weight-for-length z-scores, HC—Head Circumference, FFM—Fat-free mass, FM—Fat mass, FFMI—Fat-free mass index, FMI—Fat mass index; * Adjusted for sex, age, post-education knowledge score, morbidity and % compliance.

**Table 4:** Incidence rate ratio of overall illnesses and diarrhea.

	Episodes per Participant-Month Mean (95% CI)		Adjusted Incidence Rate Ratio (95% CI) *
	**Control**	**Intervention**	
**Overall illness**	1.5 (1.33, 1.67)	1.49 (1.33, 1.65)	0.97 (0.82, 1.16)
**Diarrhea**	0.2 (0.15, 0.25)	0.17 (0.12, 0.22)	0.85 (0.53, 1.36)

* Adjusted for sex and presented with 95% confidence intervals. Statistical significance is inferred when the confidence interval does not include 1.0.

**Table 5:** Usual nutrient intakes at baseline and endline among study groups.

	Baseline		*p*-Value	Endline		*p*-Value
	Control *N* = 58	Intervention *N* = 66		Control *N* = 55	Intervention *N* = 57	
**Energy (kcal)**	575.45 ± 115.67	576.93 ± 98.08	0.939	659.60 ± 161.33	774.33 ± 137.14	<0.001
**Protein (g)**	11.13 ± 3.80	11.02 ± 2.86	0.857	12.67 ± 4.25	16.44 ± 4.06	<0.001
**Fat (g)**	26.72 ± 5.49	26.78 ± 4.21	0.943	26.93 ± 6.11	32.26 ± 4.53	<0.001
**Carbohydrates (g)**	71.85 ± 15.82	72.28 ± 14.55	0.876	89.37 ± 26.35	102.59 ± 23.36	0.006
**Calcium (mg)**	282.17 ± 125.97	285.89 ± 110.94	0.863	255.37 ± 115.56	405.88 ± 117.32	<0.001
**Phosphorus (mg)**	164.64 ± 98.17	159.23 ± 74.15	0.733	192.39 ± 103.61	286.66 ± 94.56	<0.001
**Iron (mg)**	0.52 (0.22, 1.03)	0.64 (0.24, 0.99)	0.822	1.04 (0.48, 1.64)	1.67 (1.38, 2.42)	<0.001
**Folate (mcg)**	7.14 (2.01, 15.67)	7.67 (2.89, 13.43)	0.849	17.63 (7.49, 24.79)	14.54 (7.12, 21.95)	0.405
**Zinc (mg)**	1.15 ± 0.51	1.15 ± 0.43	0.944	1.44 ± 0.66	1.81 ± 0.62	0.003
**Vitamin A (mcg)**	9.51 (0.34, 55.77)	6.44 (0.27, 33.93)	0.735	17.87 (2.06, 54.41)	93.89 (67.81, 104.79)	<0.001
**Vitamin B6 (mg)**	0.05 (0.02, 0.13)	0.06 (0.03, 0.14)	0.582	0.15 (0.07, 0.29)	1.09 (0.77, 1.50)	<0.001
**Vitamin B12 (mcg)**	0.31 (0.31, 0.65)	0.32 (0.31, 0.62)	0.625	0.28 (0.27, 0.42)	0.42 (0.37, 0.57)	<0.001

Data reported as mean ± SD if normally distributed, and median (Q1, Q3) if otherwise. Student’s *t*-test or Mann–Whitney *U* test was applied.

**Table 6:** Dietary Diversity Score and Minimum dietary diversity from 24 h dietary recall between groups.

Variable	Baseline			Endline		
	Control (*N* = 65)	Intervention (*N* = 67)	*p*-Value	Control (*N* = 61)	Intervention (*N* = 63)	*p*-Value
**Dietary Diversity Score**	3.9 ± 1.1	3.6 ± 1.1	0.080	4.8 ± 1.4	5.3 ± 1.0	0.007
Minimum Dietary Diversity *	21 (32.3)	14 (20.9)	0.198	37 (60.7)	53 (84.1)	0.006

Data are reported as mean ± SD, and * *n* (%); Chi-square test or Fisher’s exact test was applied.

## Data Availability

Data will be made available on reasonable request to the corresponding author.

## Supplementary Materials

The following supporting information can be downloaded at: https://www.mdpi.com/article/10.3390/nu18213654/s1, Figure S1: Consort flow diagram of participant recruitment; Table S1: Nutrient composition of Milk-Legume based-Take Home Ration (ML-THR); S1: Shelf-life study; S2: Usual intake calculation [43,44,45,46]; Table S2: Group-wise monthly anthropometric measurements of study participants; Table S3: Body composition measures in the participants at baseline and endline; Table S4a: Sex-wise monthly anthropometry measurements in the participants at baseline and endline; Table S4b: Sex-wise body composition measurements in the participants at baseline and endline; Figure S2a: Weight-for-age z scores by age; Figure S2b: Weight-for-age z scores by age and sex; Figure S3a: Weight-for-length z scores by age; Figure S3b: Weight-for-length z scores by age and sex; Figure S4a: Fat-free mass index (kg/m^2^) by age; Figure S4b: Fat-free mass index (kg/m^2^) by age and sex; Table S5: Baseline characteristics between all study participants included and not included in the body composition (BC) analysis; Table S6: Difference-in-difference analysis for anthropometric and body composition measurements; Table S7: Monthly morbidity of participants; Table S8: Group-wise usual food group intake at baseline and endline; Table S9: Pre- and post-education knowledge of IYCF and WASH practices; Table S10: Comparison of IYCF and WASH knowledge category; CONSORT 2025 checklist [47].

## Abbreviations

The following abbreviations are used in this manuscript:

BC	Body composition
CFTRI	Central Food Technological Research Institute
DDS	Dietary diversity score
DIAAS	Digestible Indispensable Amino Acid Score
DID	Difference-in-differences
FFM	Fat-free mass
FM	Fat mass
FTIR	Fourier-transform infrared spectroscopy technique
HC	Head circumference
HCZ	Head circumference z-scores
IAEA	International Atomic Energy Agency
ICDS	Integrated Child Development Services
IRR	Incidence rate ratio
IYCF	Infant and Young Child Feeding practices
LAZ	Length-for-age z-scores
LMIC	Low- and middle-income countries
LMM	Linear mixed models
MDD	Minimum dietary diversity
ML-THR	Milk and legume-based Take-Home Ration
MUAC	Mid-upper arm circumference
RCT	Randomized controlled trial
SG-THR	Standard Government Take-Home Ration
SNP	Supplementary Nutrition Programme
TBW	Total Body Water
TEM	Technical error of measurement
THR	Take-Home Ration
UNICEF	United Nations Children’s Fund
WASH	Water, Sanitation and Hygiene
WAZ	Weight-for-age z-scores
WHO	World Health Organization
WLZ	Weight-for-length z-scores
