# Supplementary material for: Effect of a 3-Month Modified, High-Quality Take-Home Ration on Linear Growth and Body Composition in Rural South Indian Infants Aged 6–9 Months: A Cluster-Randomized Controlled Trial
Source: Nutrients. 2026 Nov 3;18(21):3654. doi: 10.3390/nu18213654 (PMC13765191; doi:10.3390/nu18213654)
Supplement: Supplementary file 1 [file nutrients-18-03654-s001.zip › Supplementary Materials.pdf]

S1. Shelf-life study

The ML-THR was subjected to shelf-life studies at CSIR-Central Food Technological Research Institute (CFTRI), Mysuru. CSIR-CFTRI procured the ingredients. Finger millet was washed 2-3 times and dried in a mechanical dryer maintained at 50° C for 3 to 4 hours, followed by roasting at 75° C. Peanuts were roasted at 70° C using an electric grain roaster and pulverized along with finger millet, puffed Bengal gram and jaggery. The pulverized flour was mixed with whole milk powder and oil in a planetary mixer to prepare a homogenous product. Care was taken to follow Good Manufacturing Practices to avoid any microbial contamination. The product was packed in aluminum foil and then immersed in boiling water for 8 minutes, to ensure microbial safety.

The reported critical moisture content of the ML-THR was 4.9 g/100g, and the critical relative humidity was 52%. The samples were packaged using PET/Al.Foil/PE and cast polypropylene (CPP, which can withstand temperature processing conditions), and stored under ambient (27°C, 64% relative humidity) and accelerated (38°C, 92% relative humidity) storage conditions. The stored samples were evaluated for the changes in colour, free fatty acids (FFA), sensory attributes, and microbial contents. There were no significant changes reported in colour during storage period for both the storage conditions. The FFA content in the product packaged in PET/Al/PE gradually increased over time, while a steep increase was observed for the product packed in CPP pouch after 110 days of storage period at ambient conditions. The FFA values significantly increased after storing for 80 days at accelerated conditions and the increase was higher for CPP compared to that packed in aluminum pouches. It was observed that the product stayed stable till 120 days without any deterioration in the quality characteristics at ambient conditions and for 80 days when packed in aluminum foil, at accelerated storage conditions.

Sensory profiling of the product was carried out by a panel of 8 trained analysts in CSIR-CFTRI under standardized conditions. The sensory quality showed a slight decrease in the intensity of attributes namely, fresh, nutty, leguminous, and toasted aroma during the storage period, which did not adversely affect overall storage quality of the product. However, the stored samples did not develop any noticeable off-odours or off-tastes by the end of the storage period, i.e., 120 days at ambient conditions and 90 days at accelerated conditions. Microbial analysis of the samples stored in PET/Al/PE packaging at both ambient and accelerated temperatures revealed no significant changes, and all the parameters were in compliance with the Bureau of Indian Standards specifications.

S2. Usual intake calculation

The intake distribution of study participants was obtained from the 24-hour dietary recalls of three non-consecutive days. The nutrient intakes were examined to find misreported intakes using Goldberg cutoff for the energy intake to basal metabolic rate and the outliers were removed from further analysis [43]. As the number of recall days were limited, adjustments to the distribution of observed intakes were needed to partially remove the day-to-day variability in intakes (within-person variation). When normally distributed, the usual intake was estimated using the approach proposed by the National Research Council, recommended by Institute of Medicine [44]. The general procedure for the estimation of usual intake was to fit a random effect model to the observed intake, Observed daily intake = Usual intake + deviation from usual intake.

Thus, usual intake = Group mean + $\frac{\sigma_b}{\sqrt{\sigma_b{}^2+\sigma_w{}^2/m}}$ * (Individual mean – Group mean), where $\sigma_b$is the between individual variability, σ_w is the within individual variability and 'm' is the number of recalls. The observed nutrient and food intakes from the sample were examined to check the skewness of the intake distribution and observed that all nutrients were having skewed distributions. As simple log or power transformations were not resulting in normality, a gamma regression method was used for the estimation [45]. In this method observed intake distribution was fitted with a gamma random effect model with log link as: $log\left(E\{Y_{ij}{}^*\}\right) = y_i + e_{ij}$and the estimates of group mean ($\hat{\alpha}$), within individual variability ($\hat{\sigma}_u^2$) and between individual variability ($\hat{\sigma}_y^2$) are calculated. Using $\hat{z}_l = \log\{\hat{y}_l\} = \hat{\alpha} + \frac{\hat{\sigma}_y}{\sqrt{\hat{\sigma}_y^2+\frac{\hat{\sigma}_u^2}{r}}}(\bar{z}_l - \hat{\alpha})$ the usual intake of the participants was calculated in log scale and usual intake $\hat{y}_l$ was estimated s $\exp(\hat{z}_l)$. For nutrients with zero intakes up to 5% the usual intake distribution was calculated using gamma regression method by adding 1/1000th of the mean of observed intake into each value to make the intakes to be positive.

For the nutrients or food groups which were consumed infrequently, that is when the zero intakes were 5% – 60%, a mixed distribution method was used for estimating usual intake distribution under the assumption that the quantity consumed and probability of consumption are independent [46]. In this method the positive intakes were modelled using gamma random effect model and the probability of consumption using beta-binomial regression, $f(p_i) = \frac{1}{B(\alpha,\beta)} {p_i}^{\alpha-1}(1-p_i)^{\beta-1}$, where p_i is the probability of consumption and α & β are the parameters of the beta-binomial distribution. The estimated usual intakes of positive intakes ($\hat{y}_l$) were then multiplied with the respective probability of consumption ($\hat{p}_i$) to get the final usual intake of the infrequently consumed nutrient. When the zeros in the observed intakes are more than 60%, the mixed distribution method is not appropriate and hence the usual intake was calculated from the available positive data.

43. Black, A. E. Critical evaluation of energy intake using the Goldberg cut-off for energy intake: basal metabolic rate. A practical guide to its calculation, use and limitations. *Int J Obes.* **2000**, 24(9), 1119–30, doi:10.1038/sj.ijo.0801376.
44. Institute of Medicine (US) Subcommittee on Interpretation and Uses of Dietary Reference; *Institute of Medicine (US) Standing Committee on the Scientific Evaluation of Dietary Reference Intakes. Appendix E: Adjustment of Observed Intake Data to Estimate the Distribution of Usual Intakes in a Group. In Dietary Reference Intakes: Applications in Dietary Planning*; National Academies Press (US): Washington, DC, **2003**; pp 196- 208.
45. Joseph, S.; Swaminathan, S.; Ghosh, S; Thomas, T. A New Approach to Estimate Habitual Intake of Nutrients with Skewed Distribution. *J. Nutr.* **2025**, 155, 3066-3074.

46. Joseph, S.; Ghosh, S.; Swaminathan, S.; Thomas, T. Estimation of Habitual Intake of Infrequently Consumed Nutrients Using the Mixture Distribution Method. *Front. Nutr.* **2025**, 12, 1631495, doi: 10.3389/fnut.2025.1631495.
