# Supplementary material for: Effect of a 3-Month Modified, High-Quality Take-Home Ration on Linear Growth and Body Composition in Rural South Indian Infants Aged 6–9 Months: A Cluster-Randomized Controlled Trial
Source: Nutrients. 2026 Nov 3;18(21):3654. doi: 10.3390/nu18213654 (PMC13765191; doi:10.3390/nu18213654)
Supplement: Supplementary file 1 [file nutrients-18-03654-s001.zip › Supplementary Tables.pdf]

Table S1. Nutrient composition of Milk-Legume based-Take Home Ration (ML-THR)

**Parameters**	**ML-THR (100g)**
Moisture, (% by weight)	3.10 ± 0.04
Total ash, (% by weight)	3.51 ± 0.07
Energy (kcal)	473 ± 1.5
Protein, (% by weight)	13.6 ± 0.1
Fat, (% by weight)	20.9 ± 0.1
Crude fiber, (% by weight)	1.30 ± 0.03
Carbohydrate by difference, (% by weight)	57.6 ± 0.8
Calcium (mg)	573.5 ± 3.0
Iron (mg)	2.49 ± 0.07
Zinc (mg)	1.39 ± 0.03
Phosphorous (mg)	368.1 ± 5.1
Vitamin B1 (mg)	BDL of 0.1
Vitamin B2 (mg)	0.40 ± 0.01
Vitamin B3 (mg)	13 ± 0.07
Vitamin B6 (mg)	4.14 ± 0.09
Vitamin B12 (μg)	0.30 ± 0.02
Vitamin B9 (mg)	BDL of 0.1
Vitamin A (mg)	0.20 ± 0.005
Vitamin C (mg)	24.1 ± 0.3

Abbreviation: BDL-below detectable level

The proximate analysis was conducted by CSIR-Central Food Technological Research Institute (CFTRI), Mysuru, India

Table S2. Group-wise monthly anthropometric measurements of study participants

Variable	Group	Baseline	1st Month	2nd Month	Endline
LAZ	Control	-1.17 ± 1.11 (n=66)	-1.15 ± 0.93 (n=52)	-1.26 ± 0.89 (n=53)	-1.40 ± 0.98 (n=62)
	Intervention	-1.27 ± 1.05 (n=67)	-1.23 ± 0.96 (n=58)	-1.26 ± 0.96 (n=57)	-1.38 ± 0.94 (n=64)
WAZ	Control	-1.17 ± 0.93 (n=66)	-1.21 ± 0.99 (n=64)	-1.17 ± 0.94 (n=64)	-1.28 ± 0.91 (n=63)
	Intervention	-1.07 ± 0.97 (n=67)	-0.97 ± 0.93 (n=65)	-0.98 ± 0.95 (n=63)	-1.12 ± 0.98 (n=64)
WLZ	Control	-0.59 ± 1.06 (n=65)	-0.74 ± 1.09 (n=52)	-0.75 ± 0.95 (n=53)	-0.74 ± 0.90 (n=62)
	Intervention	-0.40 ± 1.15 (n=67)	-0.29 ± 0.95 (n=58)	-0.29 ± 1.11 (n=57)	-0.58 ± 1.05 (n=64)
Length (cm)	Control	67.24 ± 2.84 (n=66)	68.52 ± 2.45 (n=52)	69.64 ± 2.34 (n=53)	70.53 ± 2.52 (n=62)
	Intervention	67.11 ± 2.51 (n=67)	68.73 ± 2.26 (n=58)	69.73 ± 2.25 (n=57)	70.93 ± 2.35 (n=64)
Weight (kg)	Control	7.30 ± 0.82 (n=66)	7.53 ± 0.88 (n=62)	7.78 ± 0.88 (n=66)	7.87 ± 0.85 (n=63)
	Intervention	7.45 ± 0.89 (n=67)	7.84 ± 0.89 (n=63)	8.00 ± 0.95 (n=64)	8.16 ± 0.98 (n=64)
HC (cm)	Control	42.31 ± 1.43 (n=67)	42.76 ± 1.23 (n=49)	43.04 ± 1.43 (n=55)	43.40 ± 1.34 (n=62)
	Intervention	42.52 ± 1.40 (n=67)	43.20 ± 1.29 (n=59)	43.78 ± 1.19 (n=51)	43.80 ± 1.37 (n=64)
MUAC (cm)	Control	13.42 ± 0.91 (n=67)	13.69 ± 0.92 (n=62)	13.80 ± 0.90 (n=66)	13.72 ± 0.95 (n=63)
	Intervention	13.67 ± 1.00 (n=67)	13.95 ± 0.92 (n=63)	14.08 ± 1.05 (n=63)	13.92 ± 0.99 (n=64)
Stunting (LAZ <-2)*	Control	13 (19.7) (n=66)	13 (25.0) (n=52)	10 (18.9) (n=53)	20 (32.3) (n=62)
	Intervention	13 (19.4) (n=67)	13 (22.4) (n=58)	14 (24.6) (n=57)	18 (28.1) (n=64)
Wasting (WLZ<-2)*	Control	4 (6.2) (n=65)	6 (11.5) (n=52)	3 (5.7) (n=53)	4 (6.5) (n=62)
	Intervention	3 (4.5) (n=67)	1 (1.7) (n=58)	1 (1.8) (n=57)	3 (4.7) (n=64)
Underweight (WAZ<-2)*	Control	13 (19.7) (n=66)	14 (21.9) (n=64)	11 (17.2) (n=64)	15 (23.8) (n=63)
	Intervention	8 (11.9) (n=67)	6 (9.2) (n=65)	9 (14.3) (n=63)	14 (21.9) (n=64)
Overweight (WLZ>-2)*	Control	0 (0.0)	0 (0.0)	0 (0.0)	0 (0.0)

		(n=65)	(n=52)	(n=53)	(n=62)
	Intervention	2 (3.0)<br>(n=67)	1 (1.7)<br>(n=58)	2 (3.5)<br>(n=57)	1 (1.6)<br>(n=64)

Abbreviation: n-number of participants, LAZ-length-for-age z-scores, WAZ-weight-for-age z-scores, WLZ-weight-for-length z- scores, HC-head circumference, MUAC-mid-upper arm circumference; Values are expressed as mean ± SD or *n (%), as appropriate. Note: n differs across variables due to missing data. Note: between group comparisons were performed for baseline and endline using independent t test or Mann-Whitney U test or chi-square (outcome was powered for these 2 times points) *Statistical Significance was defined when p value <0.05. No statistical significance was observed.

Table S3. Body composition measures in the participants at baseline and endline

Variable	Group	n	Baseline	p-value	n	Endline	p-value
Fat-free mass (kg)	Control	35	5.61 ± 0.60	0.103	28	6.20 ± 0.71	0.192
	Intervention	42	5.83 ± 0.56		40	6.41 ± 0.54	
Fat mass (kg)	Control	35	1.61 ± 0.55	0.170	28	1.81 ± 0.53	0.100
	Intervention	42	1.44 ± 0.50		40	1.57 ± 0.64	
Fat-free mass %	Control	35	78.05 ± 5.99	0.083	28	77.63 ± 5.25	0.037
	Intervention	42	80.42 ± 5.74		40	80.70 ± 6.66	
Fat mass %	Control	35	21.95 ± 5.99	0.083	28	22.37 ± 5.25	0.037
	Intervention	42	19.58 ± 5.74		40	19.30 ± 6.66	
FFMI (kg/m^2)	Control	34	12.63 ± 0.96	0.095	27	12.47 ± 1.03	0.138
	Intervention	42	13.03 ± 1.12		40	12.83 ± 0.87	
FMI (kg/m^2)	Control	34	3.65 ± 1.23	0.126	27	3.71 ± 0.97	0.042
	Intervention	42	3.22 ± 1.13		40	3.14 ± 1.28	

Abbreviation: n-number of participants; FFMI-fat-free mass index, FMI-fat mass index; Values are expressed as mean ± SD; Student's *t*-test or Mann Whitney *U* test was applied.

Table S4a. Sex-wise monthly anthropometry measurements in the participants at baseline and endline

Variable	Group	Baseline	1st Month	2nd Month	Endline
**Boys**					
LAZ	Control	-1.29 ± 1.12 (n=28)	-1.23 ± 0.91 (n=21)	-1.40 ± 0.85 (n=22)	-1.64 ± 1.08 (n=25)
	Intervention	-1.44 ± 1.08 (n=39)	-1.48 ± 0.96 (n=34)	-1.50 ± 1.00 (n=33)	-1.49 ± 0.99 (n=38)
WAZ	Control	-1.33 ± 0.96 (n=27)	-1.38 ± 0.97 (n=27)	-1.30 ± 0.95 (n=26)	-1.48 ± 0.87 (n=25)
	Intervention	-1.13 ± 1.08 (n=39)	-1.02 ± 1.03 (n=39)	-1.00 ± 1.05 (n=37)	-1.18 ± 1.07 (n=38)
WLZ	Control	-0.69 ± 1.08 (n=27)	-0.91 ± 1.15 (n=21)	-0.87 ± 0.99 (n=22)	-0.87 ± 0.97 (n=25)
	Intervention	-0.35 ± 1.24 (n=39)	-0.23 ± 1.04 (n=34)	-0.16 ± 1.21 (n=33)	-0.57 ± 1.15 (n=38)
Length (cm)	Control	68.23 ± 2.58 (n=28)	69.60 ± 2.11 (n=21)	70.59 ± 2.09 (n=22)	71.29 ± 2.59 (n=25)
	Intervention	67.54 ± 2.46 (n=39)	68.99 ± 2.21 (n=34)	69.92 ± 2.34 (n=33)	71.40 ± 2.34 (n=38)
Weight (kg)	Control	7.58 ± 0.80 (n=27)	7.82 ± 0.82 (n=25)	8.07 ± 0.82 (n=28)	8.13 ± 0.77 (n=25)
	Intervention	7.68 ± 0.94 (n=39)	8.10 ± 0.93 (n=37)	8.24 ± 1.02 (n=38)	8.39 ± 1.04 (n=38)
HC (cm)	Control	42.96 ± 1.43 (n=28)	43.35 ± 0.97 (n=18)	43.73 ± 1.50 (n=23)	44.02 ± 1.40 (n=25)
	Intervention	43.03 ± 1.26 (n=39)	43.67 ± 1.23 (n=35)	44.20 ± 1.13 (n=32)	44.31 ± 1.29 (n=38)
MUAC (cm)	Control	13.61 ± 0.98 (n=28)	13.96 ± 0.79 (n=25)	14.00 ± 0.78 (n=28)	13.94 ± 0.86 (n=25)
	Intervention	13.88 ± 1.05 (n=39)	14.10 ± 1.00 (n=37)	14.25 ± 1.09 (n=37)	14.11 ± 1.09 (n=38)
**Girls**					
LAZ	Control	-1.07 ± 1.12 (n=38)	-1.09 ± 0.96 (n=31)	-1.16 ± 0.92 (n=31)	-1.25 ± 0.89 (n=37)
	Intervention	-1.02 ± 0.97 (n=28)	-0.89 ± 0.87 (n=24)	-0.94 ± 0.81 (n=24)	-1.21 ± 0.85 (n=26)
WAZ	Control	-1.06 ± 0.91 (n=39)	-1.09 ± 0.99 (n=37)	-1.09 ± 0.94 (n=38)	-1.16 ± 0.93 (n=38)
	Intervention	-0.97 ± 0.81 (n=28)	-0.89 ± 0.77 (n=26)	-0.95 ± 0.81 (n=26)	-1.04 ± 0.84 (n=26)
WLZ	Control	-0.51 ± 1.06	-0.63 ± 1.05	-0.67 ± 0.92	-0.66 ± 0.86

		(n=38)	(n=31)	(n=31)	(n=37)
	Intervention	-0.45 ± 1.02 (n=28)	-0.37 ± 0.83 (n=24)	-0.48 ± 0.96 (n=24)	-0.59 ± 0.90 (n=26)
Length (cm)	Control	66.52 ± 2.83 (n=38)	67.79 ± 2.42 (n=31)	68.97 ± 2.31 (n=31)	70.02 ± 2.37 (n=37)
	Intervention	66.51 ± 2.49 (n=28)	68.36 ± 2.32 (n=24)	69.46 ± 2.15 (n=24)	70.24 ± 2.23 (n=26)
Weight (kg)	Control	7.10 ± 0.79 (n=39)	7.34 ± 0.88 (n=37)	7.57 ± 0.86 (n=38)	7.70 ± 0.86 (n=38)
	Intervention	7.14 ± 0.71 (n=28)	7.48 ± 0.71 (n=26)	7.65 ± 0.73 (n=26)	7.82 ± 0.78 (n=26)
HC (cm)	Control	41.85 ± 1.24 (n=39)	42.41 ± 1.24 (n=31)	42.54 ± 1.16 (n=32)	42.98 ± 1.13 (n=37)
	Intervention	41.80 ± 1.27 (n=28)	42.51 ± 1.05 (n=24)	43.07 ± 0.92 (n=19)	43.06 ± 1.16 (n=26)
MUAC (cm)	Control	13.29 ± 0.85 (n=39)	13.50 ± 0.97 (n=37)	13.66 ± 0.96 (n=38)	13.57 ± 0.99 (n=38)
	Intervention	13.38 ± 0.86 (n=28)	13.72 ± 0.77 (n=26)	13.85 ± 0.96 (n=26)	13.65 ± 0.75 (n=26)

Abbreviation: n-number of participants, LAZ-length-for-age z-scores, WAZ- weight-for-age z-scores, WLZ- weight-for-length z- scores, HC-head Circumference, MUAC-mid-upper arm circumference. Note: n differs across variables due to missing data. Note: between group comparisons were performed for baseline and endline using independent t test or Mann-Whitney U test (outcome was powered for these 2 times points) *Statistical Significance was defined when p value <0.05. No statistical significance was observed.

Table S4b. Sex-wise body composition measurements in the participants at baseline and endline

Variable	Group	n	Baseline	p-value	n	Endline	p- value
**Boys**							
Fat-free mass (kg)	Control	12	5.85 ± 0.47	0.589	9	6.48 ± 0.40	0.449
	Intervention	23	5.95 ± 0.52		20	6.61 ± 0.49	
Fat mass (kg)	Control	12	1.47 ± 0.57	0.878	9	1.88 ± 0.52	0.073
	Intervention	23	1.44 ± 0.47		20	1.49 ± 0.52	
Fat-free mass %	Control	12	80.41 ± 6.05	0.894	9	77.77 ± 4.19	0.034
	Intervention	23	80.69 ± 5.35		20	81.91 ± 5.11	
Fat mass %	Control	12	19.59 ± 6.05	0.894	9	22.23 ± 4.19	0.034
	Intervention	23	19.31 ± 5.35		20	18.09 ± 5.11	
FFMI (kg/m^2)	Control	12	13.10 ± 0.72	0.931	9	13.01 ± 0.64	0.933
	Intervention	23	13.13 ± 1.10		20	12.98 ± 0.98	
FMI (kg/m^2)	Control	12	3.27 ± 1.19	0.839	9	3.77 ± 0.99	0.045
	Intervention	23	3.18 ± 1.04		20	2.91 ± 0.98	
**Girls**							
Fat-free mass (kg)	Control	23	5.48 ± 0.62	0.289	19	6.07 ± 0.80	0.516
	Intervention	19	5.69 ± 0.59		20	6.21 ± 0.52	
Fat mass (kg)	Control	23	1.68 ± 0.54	0.164	19	1.77 ± 0.55	0.579
	Intervention	19	1.44 ± 0.54		20	1.66 ± 0.74	
Fat-free mass %	Control	23	76.82 ± 5.70	0.090	19	77.56 ± 5.79	0.386
	Intervention	19	80.08 ± 6.31		20	79.49 ± 7.86	
Fat mass %	Control	23	23.18 ± 5.70	0.090	19	22.44 ± 5.79	0.386
	Intervention	19	19.92 ± 6.31		20	20.51 ± 7.86	
FFMI (kg/m^2)	Control	22	12.37 ± 0.99	0.120	18	12.20 ± 1.09	0.125
	Intervention	19	12.91 ± 1.17		20	12.68 ± 0.74	
FMI (kg/m^2)	Control	22	3.86 ± 1.23	0.141	18	3.68 ± 0.98	0.463
	Intervention	19	3.27 ± 1.25		20	3.37 ± 1.51	

Abbreviation: n-number of participants, FFMI-fat-free mass index, FMI-fat mass index; Values are expressed as mean ± SD; Student's *t*-test or Mann-Whitney U test was applied.

Table S5. Baseline characteristics between all study participants included and not included in the body composition (BC) analysis

Variables	Included in BC analysis	Not included in BC analysis
**N**	77	56
**Boys, n (%)**	33 (42.9)	32 (57.1)
**Site (*Sub-district*), n (%)**		
Site 1 (*Hiriyur*)	52 (67.5)	41 (73.2)
Site 2 (*Molkalmuru*)	25 (32.5)	15 (26.8)
**Age (months)***	7.71 ± 0.67	7.80 ± 0.52
**Birthweight (kg)***	2.82 ± 0.50	2.81 ± 0.45
**Birth order of the participant, n (%)**		
1	45 (58.4)	21 (37.5)
2	22 (28.6)	26 (46.4)
>=3	10 (13.0)	9 (16.1)
**Mother's education, n (%)**		
Illiterate	9 (11.7)	7 (12.5)
Primary School (1st - 5th std)	6 (7.8)	3(5.4)
Middle School (6th - 8th std)	7 (9.1)	4 (7.1)
High School (9th - 10th std)	22 (28.6)	17 (30.4)
PUC/Diploma	26 (33.8)	21 (37.5)
>=Graduation	7 (9.1)	4 (7.1)
**Monthly Income (INR)***	10409.09 ± 5597.65	11491.07 ± 8742.52
**#Socioeconomic class, n (%)**		
Upper	16 (20.8)	8 (14.3)
Upper middle	4 (5.2)	2 (3.6)
Lower middle	20 (26.0)	21 (37.5)
Upper lower	35 (45.5)	25 (44.6)
Lower	2 (2.6)	0 (0.0)
**Household food security status, n (%)**		
Secure	68 (88.3)	48 (85.7)
Mildly insecure	4 (5.2)	1 (1.8)
Moderately insecure	3 (3.9)	1 (1.8)
Severely insecure	2 (2.6)	6 (10.7)
**No. of breastfed infants, n (%)**	77 (100.0)	56 (100)
**Initiation of complementary foods (age in months)***	6.53 ± 1.08	6.61 ± 0.87
**Quantity of THR received***		
Pushti (kg)	2.96 ± 0.20	2.92 ± 0.36
Jaggery (kg)	1.14 ± 0.30	1.19 ± 0.25
Whole milk powder (g)	303.33 ± 104.08	329.81 ± 145.61
Broken rice (kg)	2.08 ± 0.05	2.08 ± 0.04
Whole mung bean (g)	467.25 ± 188.50	412.40 ± 88.10
Split mung bean (g)	587.25 ± 108.50	533.00 ± 0.00

**Caregiver reported infant SG-THR use, n (%)**	59 (76.6)	44 (78.6)
**Consumption of SG-THR, n (%)**		
Only by infant	34 (44.2)	24 (42.9)
Shared among siblings	1 (1.3)	0 (0.0)
Shared among family members	24 (31.2)	20 (35.7)
Anthropometric measurements*		
Length-for-age z-scores (LAZ)	-1.34 ± 1.03	-1.03 ± 1.13
Weight-for-age z-scores (WAZ)	-1.24 ± 0.91	-0.93 ± 0.99
Weight-for length z-scores (WLZ)	-0.57 ± 1.05	-0.37 ± 1.19
Head circumference z-scores	-1.29 ± 0.98	-1.20 ± 0.95
Mid upper arm circumference z-scores	-0.73 ± 0.84	-0.54 ± 0.92
Stunting (LAZ <-2), n (%)	16 (21.1)	9 (16.1)
Wasting (WLZ<-2), n (%)	4 (5.3)	3 (5.5)
Underweight (WAZ<-2), n (%)	15 (19.5)	6 (10.9)
Overweight (WLZ>-2), n (%)	1 (1.3)	1 (1.8)
Blood parameter*		
N	75	49
Haemoglobin (g/dl)	10.17 ± 1.21	10.35 ± 1.17

Abbreviation: N-number of participants, PUC-Pre-university college, SG-THR- Standard Government Take-Home Ration; Values are expressed as *mean ± SD

Table S6. Difference-in-difference analysis for anthropometric and body composition measurements

Variable	Group	n	Mean difference over 3 months (95% CI)	DID (95% CI)	p-value between group
**LAZ**	Control	62	-0.25 (-0.42, -0.08)	0.16 (-0.06, 0.38)	0.164
	Intervention	64	-0.09 (-0.24, 0.05)		
**WAZ**	Control	62	-0.13 (-0.22, -0.04)	0.06 (-0.06, 0.18)	0.322
	Intervention	64	-0.07 (-0.15, 0.01)		
**WLZ**	Control	61	-0.16 (-0.33, 0.01)	-0.04 (-0.28, 0.19)	0.714
	Intervention	64	-0.21 (-0.36, -0.05)		
**HCZ**	Control	63	-0.10 (-0.22, 0.01)	0.12 (-0.03, 0.27)	0.118
	Intervention	64	0.01 (-0.08, 0.11)		
**Length (cm)**	Control	62	3.31 (2.92, 3.7)	0.54 (0.04, 1.05)	0.037
	Intervention	64	3.85 (3.53, 4.18)		
**Weight (kg)**	Control	61	0.58 (0.49, 0.66)	0.12 (0.01, 0.23)	0.041
	Intervention	64	0.69 (0.62, 0.77)		
**HC (cm)**	Control	62	1.08 (0.95, 1.21)	0.19 (0.01, 0.38)	0.039
	Intervention	64	1.28 (1.15, 1.4)		
**FFM (kg)**	Control	25	0.51 (0.4, 0.62)	0.12 (-0.04, 0.28)	0.140
	Intervention	37	0.63 (0.51, 0.75)		
**FM (kg)**	Control	25	0.07 (-0.04, 0.17)	0.02 (-0.17, 0.21)	0.849
	Intervention	37	0.08 (-0.07, 0.24)		
**FFM %**	Control	25	0.79 (-0.43, 2.02)	0.03 (-2.19, 2.25)	0.976
	Intervention	37	0.83		

			(-1.02, 2.68)		
**FM %**	Control	25	-0.79<br>(-2.02, 0.43)	-0.03<br>(-2.25, 2.19)	0.976
	Intervention	37	-0.83<br>(-2.68, 1.02)		
**FFMI (kg/m^2)**	Control	24	0.00<br>(-0.29, 0.28)	-0.16<br>(-0.58, 0.26)	0.467
	Intervention	37	-0.16<br>(-0.47, 0.15)		
**FMI (kg/m^2)**	Control	24	-0.14<br>(-0.35, 0.08)	-0.05<br>(-0.43, 0.33)	0.800
	Intervention	37	-0.19<br>(-0.5, 0.13)		

Note: Abbreviation: n-number of participants, DID-difference-in-difference, LAZ-Length-for-age z-scores, WAZ-Weight-for-age z-scores, WLZ-Weight-for-length z-scores, HC-Head Circumference, MUAC-Mid-upper arm circumference, FFM-Fat-free mass, FM-Fat mass, FFMI- Fat-free mass index, FMI-Fat mass index; Values are expressed as mean difference (95% CI) and DID (95% CI).

Table S7. Monthly morbidity of participants

	Control n/total (%)	Intervention n/total (%)	p-value
**Diarrhea**			
Baseline	7/64 (10.9)	12/65 (18.5)	0.338
1st month	13/64 (20.3)	11/64 (17.2)	0.821
2nd month	12/66 (18.2)	12/64 (18.8)	1.000
Endline	19/62 (30.6)	9/63 (14.3)	0.048*
**Abdominal pain**			
Baseline	2/64 (3.1)	0/65 (0.0)	0.244
1st month	2/64 (3.1)	0/64 (0.0)	0.496
2nd month	1/66 (1.5)	0/64 (0.0)	1.000
Endline	0/62 (0.0)	0/63 (0.0)	0.929
**Vomiting**			
Baseline	0/64 (0.0)	0/65 (0.0)	0.930
1st month	6/64 (9.4)	2/64 (3.1)	0.273
2nd month	3/66 (4.5)	6/64 (9.4)	0.321
Endline	7/62 (11.3)	2/63 (3.2)	0.095
**Coughing**			
Baseline	8/64 (12.5)	17/65 (26.2)	0.082
1st month	24/64 (37.5)	29/64 (45.3)	0.473
2nd month	26/66 (39.4)	26/64 (40.6)	1.000
Endline	30/62 (48.4)	36/63 (57.1)	0.423
**Runny nose**			
Baseline	11/64 (17.2)	21/65 (32.3)	0.066
1st month	38/64 (59.4)	34/64 (53.1)	0.593
2nd month	37/66 (56.1)	32/64 (50.0)	0.404
Endline	45/62 (72.6)	47/63 (74.6)	0.957
**Breathing difficulty**			
Baseline	0/64 (0.0)	1/65 (1.5)	1.000
1st month	0/64 (0.0)	1/64 (1.6)	1.000
2nd month	1/66 (1.5)	1/64 (1.6)	1.000
Endline	0/62 (0.0)	2/63 (3.2)	0.496
**Ear pain**			
Baseline	3/64 (4.7)	0/65 (0.0)	0.119
1st month	1/64 (1.6)	0/64 (0.0)	1.000
2nd month	0/66 (0.0)	0/64 (0.0)	0.861
Endline	0/62 (0.0)	2/63 (3.2)	0.496

**Sore throat**			
Baseline	0/64 (0.0)	0/65 (0.0)	0.930
1st month	1/64 (1.6)	0/64 (0.0)	1.000
2nd month	0/66 (0.0)	0/64 (0.0)	0.861
Endline	0/62 (0.0)	1/63 (1.6)	1.000
**Fever/ Tiredness**			
Baseline	15/64 (23.4)	10/65 (15.4)	0.350
1st month	24/64 (37.5)	16/64 (25)	0.182
2nd month	22/66 (33.3)	21/64 (32.8)	1.000
Endline	26/62 (41.9)	30/63 (47.6)	0.646
**Sputum/Slime during cough**			
Baseline	0/64 (0.0)	1/65 (1.5)	1.000
1st month	0/64 (0.0)	1/64 (1.6)	1.000
2nd month	0/66 (0.0)	0/64 (0.0)	0.861
Endline	1/62 (1.6)	0/63 (0.0)	0.496
**Hospitalization**			
Baseline	0/64 (0.0)	0/65 (0.0)	0.930
1st month	0/64 (0.0)	1/64 (1.6)	1.000
2nd month	1/65 (1.5)	1/64 (1.6)	1.000
Endline	1/62 (1.6)	2/63 (3.2)	1.000

Values are expressed as n/total (%); Chi-square or Fisher's exact test was applied.

Table S8. Group-wise usual food group intake at baseline and endline

	**Baseline**		**p-value**	**Endline**		**p-value**
	Control N=58	Intervention N=66		Control N=55	Intervention N=57	
Cereals (g)	20.55 ± 16.92<br>16.05<br>(9.36, 26.52)	21.81 ± 15.59<br>18.44<br>(10.44, 31.50)	0.463	45.27 ± 30.41<br>43.28<br>(22.25, 65.20)	50.35 ± 23.63<br>45.99<br>(35.89, 62.86)	0.327
Legumes (g)	0.95 ± 2.34<br>0.06<br>(0.00, 0.94)	0.35 ± 0.65<br>0.00<br>(0.00, 0.44)	0.145	1.92 ± 2.95<br>0.84<br>(0.21, 2.02)	2.30 ± 1.79<br>2.12<br>(1.32, 2.76)	0.001
Vegetables (g)	0.15 ± 0.44<br>0.00<br>(0.00, 0.16)	0.29 ± 0.81<br>0.00<br>(0.00, 0.24)	0.612	1.29 ± 2.04<br>0.47<br>(0.00, 1.81)	0.94 ± 1.54<br>0.37<br>(0.00, 0.82)	0.604
Green leafy vegetables (g)	0.29 ± 0.74<br>0.00<br>(0.00, 0.15)	0.45 ± 1.61<br>0.00<br>(0.00, 0.24)	0.913	2.60 ± 9.59<br>0.00<br>(0.00, 0.83)	0.53 ± 1.89<br>0.00<br>(0.00, 0.13)	0.084
Roots and tubers (g)	0.33 ± 0.83<br>0.00<br>(0.00, 0.25)	0.46 ± 1.38<br>0.00<br>(0.00, 0.36)	0.869	3.54 ± 13.52<br>0.63<br>(0.00, 2.58)	1.04 ± 1.28<br>0.48<br>(0.00, 1.48)	0.297
Nuts (g)	0.55 ± 1.43<br>0.00<br>(0.00, 0.18)	0.29 ± 0.71<br>0.00<br>(0.00, 0.18)	0.943	1.23 ± 1.70<br>0.56<br>(0.00, 1.81)	1.79 ± 1.34<br>1.69<br>(0.84, 2.21)	0.001
Fruits (g)	12.85 ± 22.66<br>0.46<br>(0.00, 15.87)	8.70 ± 18.53<br>0.00<br>(0.00, 4.15)	0.038	15.44 ± 27.94<br>1.13<br>(0.07, 16.99)	18.33 ± 47.61<br>0.53<br>(0.03, 16.03)	0.398
Egg (g)	0.02 ± 0.12<br>0.00<br>(0.00, 0.00)	0.00 ± 0.00<br>0.00<br>(0.00, 0.00)	0.133	1.26 ± 6.13<br>0.00<br>(0.00, 0.00)	2.99 ± 9.17<br>0.00<br>(0.00, 0.00)	0.083
Milk and milk products (g)	45.86 ± 84.63<br>0.03<br>(0.00, 45.93)	47.77 ± 76.17<br>1.52<br>(0.00, 81.12)	0.351	37.09 ± 79.19<br>0.13<br>(0.00, 38.26)	37.33 ± 70.64<br>13.19<br>(5.16, 23.82)	<0.001
Flesh foods (g)	0.00 ± 0.00<br>0.00<br>(0.00, 0.00)	0.15 ± 1.23<br>0.00<br>(0.00, 0.00)	0.357	0.60 ± 2.44<br>0.00<br>(0.00, 0.00)	0.74 ± 3.80<br>0.00<br>(0.00, 0.00)	0.935

Data represented as mean ± SD and median (Q1, Q3); Student’s t-test or Mann-Whitney U test was applied.

Table S9. Pre- and post-education knowledge of IYCF and WASH practices

	All participants' caregivers	p-value[a]	Control n (%)	p-value Within group[a]	Intervention n (%)	p-value Within group[a]
**N**	**124**		**62**		**62**	
**IYCF**						
Pre-education	7.83 ± 1.46	<0.001	7.74 ± 1.47	<0.001	7.92 ± 1.45	<0.001
Post-education	9.85 ± 0.79		9.79 ± 0.73		9.90 ± 0.84	
**WASH**						
Pre-education	10.02 ± 1.46	<0.001	9.84 ± 1.57	<0.001	10.21 ± 1.32	<0.001
Post-education	11.41 ± 0.92		11.47 ± 0.97		11.35 ± 0.87	
**Total score**						
Pre-education	17.85 ± 2.30	<0.001	17.58 ± 2.39	<0.001	18.13 ± 2.18	<0.001
Post-education	21.26 ± 1.23		21.26 ± 1.25		21.26 ± 1.21	

Values represented as mean ± SD; [a]Paired t-test

Table S10. Comparison of IYCF and WASH knowledge category

Domain	Knowledge category	Overall Caregivers n (%)		Control n (%)		Intervention n (%)		Between group p-value (Endline)[a]
		Baseline (n=132)	Endline (n=125)	Baseline (n=66)	Endline (n=62)	Baseline (n=66)	Endline (n=63)	
IYCF	Poor	8 (6.1)	0 (0.0)	4 (6.1)	0 (0.0)	4 (6.1)	0 (0.0)	1.000
	Fair	75 (56.8)	4 (3.2)	39 (59.1)	2 (2.3)	36 (54.6)	2 (3.2)	
	Good	49 (37.1)	121 (96.8)	23 (34.9)	60 (96.8)	26 (39.4)	61 (96.8)	
Within-group p value[b]		<0.001		<0.001		<0.001		
WASH	Poor	2 (1.5)	0 (0.0)	1 (1.5)	0 (0.0)	1 (1.5)	0 (0.0)	0.838
	Fair	69 (52.3)	20 (16.0)	38 (57.6)	9 (14.5)	31 (47.0)	11 (17.5)	
	Good	61 (46.2)	105 (84.0)	27 (40.9)	53 (85.5)	34 (51.5)	52 (82.5)	
Within-group p value[b]		<0.001		<0.001		<0.001		
IYCF+ WASH combined	Poor	3 (2.3)	0 (0.0)	2 (3.0)	0 (0.0)	1 (1.5)	0 (0.0)	1.000
	Fair	89 (67.4)	8 (6.4)	44 (66.7)	4 (6.5)	45 (68.2)	4 (6.4)	
	Good	40 (30.3)	117 (93.6)	20 (30.3)	58 (93.6)	20 (30.3)	59 (93.7)	
Within-group p value[b]		<0.001		<0.001		<0.001		

Values are expressed as n (%); [a] Chi-square test or Fisher's exact test; [b]McNemar's test
